# Comparison of Xpert MTB/RIF with ProbeTec ET DTB and COBAS TaqMan MTB for direct detection of *M*. *tuberculosis* complex in respiratory specimens

**DOI:** 10.1186/1471-2334-13-280

**Published:** 2013-06-20

**Authors:** Uladzimir Antonenka, Sabine Hofmann-Thiel, Laziz Turaev, Ainura Esenalieva, Mokhonim Abdulloeva, Evgeni Sahalchyk, Tarig Alnour, Harald Hoffmann

**Affiliations:** 1 IML red, Supranational Reference Laboratory of Tuberculosis, Gauting, Germany; 2 Synlab MVZ Gauting, Gauting, Germany; 3 National Institute of Tuberculosis, National Reference Laboratory of Tuberculosis, Tashkent, Republic of Uzbekistan; 4 National Reference Laboratory of Tuberculosis, Bishkek, Republic of Kyrgyzstan; 5 National Reference Laboratory of Tuberculosis, Machiton, Republic of Tajikistan; 6 Alzaeim Alazhari University, Khartoum, Sudan

**Keywords:** Tuberculosis, *Mycobacterium Tuberculosis* Complex, Nucleic Acid Amplification, Xpert MTB/RIF

## Abstract

**Background:**

Nucleic acid amplification assays allow for the rapid and accurate detection of *Mycobacterium tuberculosis* (MTB) directly in clinical specimens thereby facilitating diagnosis of tuberculosis (TB). With the fully automated Xpert MTB/RIF system (Cepheid) an innovative solution of TB diagnostics has been launched. We performed a direct head-to-head comparison of Xpert MTB/RIF with two widely used commercial assays, ProbeTec ET DTB (DTB) (Becton-Dickinson) and COBAS TaqMan MTB (CTM-MTB) (Roche).

**Methods:**

121 pre-characterized respiratory specimens (68 culture-positive for MTB complex, 24 culture-positive for non-tuberculous mycobacteria and 29 culture-negative) taken from our frozen specimen bank were tested for the presence of MTB complex by the three assays.

**Results:**

Among culture-positive samples (n = 68), overall sensitivity for detection of MTB complex was 74.6%, 73.8%, and 79.1% for Xpert MTB/RIF, CTM-MTB, and DTB, respectively. Within the subgroup of smear-negative TB samples (n = 51) sensitivity was 68% for Xpert MTB/RIF and CTM-MTB and 72% for DTB. Among smear-positive TB samples (n = 17), all (100%) were detected by DTB and 94.1% and 93.3% by Xpert MTB/RIF and CTM-MTB, respectively. Specificity was best for CTM-MTB (100%) and lowest for Xpert MTB/RIF (96.2%) due to misidentification of two NTM samples as MTB complex. CTM-MTB yielded the highest rate of invalid results (4.1%) (0.8% by Xpert MTB/RIF and DTB, respectively).

**Conclusions:**

The direct comparison of Xpert MTB/RIF with CTM-MTB and DTB yielded similar overall performance data. Whereas DTB was slightly superior to Xpert MTB/RIF in terms of sensitivity, at least in the sample collection tested here, CTM-MTB performed best in terms of specificity.

## Background

The introduction of nucleic acid amplification tests (NAAT) for detection of *M*. *tuberculosis* complex (MTBC) directly from clinical specimens has greatly improved tuberculosis (TB) diagnostics by giving results within one day. Such tests confirm the diagnosis of TB more reliably and with higher sensitivity than smear microscopy. Moreover, their high positive predictive values allow discriminating between MTB and non-tuberculous mycobacteria in smear positive specimens.

A number of commercial assays are available which use different molecular approaches to amplify and detect MTBC. These are, for example, COBAS TaqMan MTB (CTM-MTB) (Roche), Amplified *M*. *tuberculosis* Direct (AMTD) (GenProbe), ProbeTec ET DTB (DTB) (Becton-Dickinson), *Mycobacterium tuberculosis* Ligase Chain reaction (LCx) (Abott) or GenoType Mycobacteria Direct assay (GTMD) (HAIN Lifesciences). Following a large multi-country evaluation published in 2010 [1], the World Health Organization (WHO) endorsed a new NAAT as innovative solution of rapid TB diagnostics especially in settings of high-prevalence of HIV-associated disease and/or MDR-TB. The Xpert MTB/RIF (Cepheid) represents the first assay which uses a fully integrated real-time PCR platform allowing the simultaneous detection of MTBC and resistance to rifampin (RMP) based on *rpoB* as target sequence [2]. The cartridge-based system is easy to use without need of prior sputum processing, bio-safe and bears a minimized risk of cross-contamination. Both, a first multi-country evaluation study [1] as well as a consecutive multi-centre implementation study [3] showed good specificity (99.2%) and excellent sensitivity values (92.2% and 90.3%, respectively), in particular for smear-negative TB specimens (72.5% and 76.9%, respectively). These data suggested that sensitivity might be superior to that of other commercial assays [4-7].

Indirect comparison of Xpert MTB/RIF with other assays by reviewing data from evaluation studies is difficult due to significant differences in the study design and the sample collection. Comparative analysis is further hampered by the fact that published evaluation data for Xpert MTB/RIF appeared very heterogeneous, for example, there was a high variation in sensitivity for smear-positive (97.7% - 100%) or smear-negative pulmonary TB (43.4% - 75.3%) in different studies [2,8-11]. So far, studies on the direct comparison of Xpert MTB/RIF with other commercial assays are rare. Teo et al. [12] reported the comparison of Xpert MTB/RIF with AMTD using 162 respiratory and non-respiratory samples; Causse et al. [13] compared Xpert MTB/RIF to CTM-MTB using 340 non-respiratory specimens. Some other studies compared performance of Xpert MTB/RIF to that of in-house real-time PCR assays [14,15].

The aim of the present study was the direct head-to-head comparison of Xpert MTB/RIF with two widely used commercial NAATs, i.e. CTM-MTB and DTB. CTM-MTB uses real-time PCR technology like Xpert MTB/RIF but is based on detection of 16S rDNA sequences. The COBAS TaqMan system has recently replaced the well-established COBAS Amplicor MTB which has been proven highly specific [4-6]. The DTB assay, on the other hand, is based on strand-displacement amplification (SDA) technique detecting IS*6110* target sequences. Due to the multi-copy nature of this genetic element, DTB is assumed to perform better in terms of sensitivity. Using a set of 121 decontaminated specimens, the three assays were compared head-to-head. In a retrospective setting we paid special attention to (i) culture-positive smear-negative TB samples on one hand to compare sensitivity with paucibacillary TB samples and (ii) non-TB samples showing growth of NTM in order to compare the accuracy of the NAATs regarding discrimination between MTBC and NTM.

## Methods

### Specimens

One hundred twenty-one clinical specimens (68 culture-positive for MTBC, 24 culture-positive for NTM and 29 culture-negative), frozen in sediment, were taken from our pre-characterized specimen bank at the IML Gauting. All specimens had been received for routine mycobacterial testing from June 2007 to January 2011 and originated from 121 patients with suspected MTBC or NTM infection. Specimens were selected with a focus on smear-negative TB cases and NTM samples. The study protocol involving the use of clinical specimens and human data has been approved by the ethics committees of the Bayrische Landesärztekammer (no. 06043) and the University of Munich (no. 437–12). The clinical specimens comprised respiratory samples only (106 sputa, 15 bronchial aspirates). Of 92 culture-positive samples, 66 were positive for MTB, one for *M*. *africanum*, one for *M*. *bovis* spp. *bovis* and from 24 samples grew NTM (six *M*. *avium*, five *M*. *intracellulare*, five *M*. *kansasii*, four *M*. *xenopi*, three *M*. *malmoense* and one *M*. *abscessus*).

### Specimen processing and culturing

All specimens were processed by standard *N*-acetyl-*L*-cysteine and sodium hydroxide (NALC-NaOH) according to DIN 58943–3 [16]. After a centrifugation step, the sediment was resuspended with 1.0 ml phosphate buffer (pH 6.8) and inoculated in one fluid medium (MGIT™, Becton-Dickinson, Heidelberg, Germany) and two solid media (Loewenstein-Jensen, Stonebrinck). Additionally, smears were prepared and stained using Auramin for the detection of acid-fast bacilli (AFB). The remainder of the decontaminated material (approximately 0.8 to 1.2 ml) was frozen at −80°C in our specimen bank for potential later research purposes. When cultures turned positive, isolates were differentiated using the DNA strip assays, i.e. Genotype CM and/or MTBC assays (HAIN Lifescience GmbH, Nehren, Germany). Drug susceptibility testing of MTBC isolates for RMP was done using the Bactec MGIT 960 with a final concentration of 1 μg/ml according to the manufacturer’s instructions (Becton-Dickinson, Heidelberg, Germany). Cultures were considered negative when no growth has occurred after incubation for 8 weeks.

### NAAT

Decontaminated specimens were taken from our frozen archive, filled up with normal saline to a final volume of 1.5 ml and thoroughly mixed. From one tube, samples were split into aliquots for further processing according to Xpert MTB/RIF (500 μl), DTB (500 μl) and CTB-MTB (100 μl). Thus, identical conditions existed for all three tests. Specimens were not refrozen before further analysis. All tests were performed according to the manufacturer’s instruction and are detailed below.

For Xpert MTB/RIF, 1.5 ml sample reagent was added to 500 μl decontaminated specimen. After 15 min of incubation with intermittent shaking, 2 ml of the liquefied inactivated sample was added to the cartridge and placed into the instrument for automated processing, amplification and detection. Results of real-time PCR were given as “MTB not detected”, “MTB detected” (classified by the instrument as “high”, “medium”, “low” or “very low”), “invalid”, “error” or “no result”, and RMP susceptible or resistant.

For DTB, 500 μl of decontaminated specimen was washed in 1 ml washing buffer, centrifuged and inactivated at 105°C for 30 min. Further lysis was done by sonication at 65°C for 45 min. Samples (150 μl) were transferred into priming microwell plates (containing primers, probes and internal controls) for priming reactions. Samples from each priming well were then transferred into corresponding wells on amplification microwell plates (containing DNA polymerase and restriction endonuclease), sealed and immediately placed into the BD ProbeTec ET instrument for SDA. The instrument reported amplification signals > 3.500 method-other-than-acceleration (MOTA) units as positive.

For COBAS TaqMan MTB, 100 μl of decontaminated specimen was washed, centrifuged and lysed at 60°C for 45 min. PCR mixes were prepared, the DNA was added and tubes were placed into the COBAS TaqMan 48 analyzer for real-time PCR. Cycle threshold values (CT) for both, DNA of MTBC and controls, were determined by the TaqMan 48 analyzer and results were given as “MTB complex not detected” or “MTB complex positive” together with the cycle number at which the signal intensities reached threshold levels.

### Analysis of results

Conventional culture was considered the “gold standard” for evaluation of the NAATs. In case of discrepant results between NAAT and culture or in case of invalid results, no repeat testing was carried out. Since repeat testing was generally not possible for Xpert MTB/RIF due to lack of residual decontaminated material, we proceeded in the same manner for all three assays. Invalid results or tests without interpretable results were excluded from calculation of sensitivity and specificity. Sensitivity and specificity of the assays with 95% confidence intervals were determined using the Vassar-Stats calculator. Statistical tests were done using uncorrected chi-square test or Mid-P exact test (two-tailed) using OpenEpi version 2.3. A *P* value of <0.05 was considered statistically significant.

## Results

Specimens included in the study comprised 68 (56%) culture-positive TB samples of which 17 (25%) were AFB smear-positive and 51 (75%) smear-negative (Table 1). Fifty-three (44%) comprised non-TB samples. Of these, 29 (55%) were culture-negative/smear-negative and from 24 (45%) grew NTM. All but one of the NTM samples were AFB smear-positive.

**Table 1:** **Results from Xpert MTB**/**RIF**, **CTM**-**MTB and DTB**

	**Numbers of samples with indicated test results**								
	**Xpert MTB/RIF**			**CTM-MTB**			**DTB**		
	**Pos**	**Neg**	**Inv**	**Pos**	**Neg**	**Inv**	**Pos**	**Neg**	**Inv**
TB	50	17	1	48	17	3	53	14	1
*MIC pos*, *Cx pos*	*16*	*1*	*0*	*14*	*1*	*2*	*17*	*0*	*0*
*MIC neg*, *Cx pos*	*34*	*16*	*1*	*34*	*16*	*1*	*36*	*14*	*1*
no TB	2	51	0	0	51	2	1	52	0
*MIC neg*, *Cx neg*	*0*	*29*	*0*	*0*	*28*	*1*	*0*	*29*	*0*
*NTM*	*2*	*22*	*0*	*0*	*23*	*1*	*1*	*23*	*0*
TOTAL	52	68	1	48	68	5	54	66	1

*Cx* culture (fluid or solid media), *MIC* smear microscopy, *TB* tuberculosis, *NTM* non-tuberculous mycobacteria, *neg* negative, *pos* positive, *inv* invalid test result.

Overall, sensitivity values for detection of MTBC in culture positive specimens (n = 68) were 74.6%, 73.8%, and 79.1% for Xpert MTB/RIF, CTM-MTB, and DTB, respectively (Tables 1 and 2). Among the 17 culture-positive/smear-positive TB samples, 16 (94.1%) were positive by Xpert MTB/RIF. The CTM-MTB identified 14 (93.3%) samples whereas two of 17 samples yielded invalid results (and were not included in calculation of sensitivity). Only DTB yielded positive results for all 17 (100%) samples. The smear-positive TB sample which was not detected by Xpert MTB/RIF was of scanty grade in smear microscopy. However, both other assays yielded clearly positive results (MTB-CTM: target positive at cycle number 37.6; DTB: MOTA 80,824). The smear-positive TB sample which was missed by CTB-MTB was also of scanty grade and yielded good signals with DTB (MOTA 64,719) and weak signals with Xpert MTB/RIF (“MTB detected very low”).

**Table 2:** **Sensitivities and specificities of Xpert MTB**/**RIF**, **CTM**-**MTB and DTB**

	**% sensitivity (95% CI)**			**% specificity (95% CI)**
	**All**	**MIC pos**	**MIC neg**	
	**Cx pos**	**Cx pos**	**Cx pos**	**no TB**
Xpert MTB/RIF	74.6	94.1	68.0	96.2
	(62.2-84.1)	(69.2-99.7)	(53.1-80.0)	(85.9-99.3)
CTM MTB	73.8	93.3	68.0	100
	(61.2-83.6)	(66.0-99.6)	(53.1-80.0)	(91.2-100)
DTB	79.1	100	72.0	98.1
	(67.1-87.7)	(77.0-100)	(57.2-83.3)	(88.6-99.9)

*Cx* culture (fluid or solid media), *MIC* smear microscopy, *CI* confidence interval.

Within the group of 51 culture-positive/smear-negative TB samples, one sample yielded indeterminate results with both, Xpert MTB/RIF and DTB; another one with CTM-MTB (Table 1). Among samples with valid results, both, Xpert MTB/RIF and CTM-MTB identified 34 (68%), while DTB identified 36 (72%) (Tables 1 and 2). Twenty-six samples yielded concordantly positive MTBC results by all three assays. Three samples were identified by Xpert MTB/RIF only, two samples by DTB only, and another two by CTM-MTB only. Four samples were missed by Xpert MTB/RIF only, three by CTM-MTB only and one solely by DTB. Eight samples were detected by neither assay.

Of 53 non-TB samples, Xpert MTB/RIF identified 51 as negative specificity: 96.2% (Tables 1 and 2). DTB and CTM-MTB yielded specificity values of 98.1% and 100%, respectively. Within the sub-group of culture negative samples (n = 29), no false positive results occurred with all three assays (specificity: 100%). Within in the sub-group of culture positive samples with NTM (n = 24), Xpert MTB/RIF produced two (8.4%) false-positive MTBC results. Both samples were AFB smear-positive; from one grew *M*. *avium*, from the other one *M*. *kansasii*. One false-positive result was obtained with the DTB assay; the culture of this sample grew *M*. *xenopi*. All (100%) NTM samples were correctly identified as MTBC negative by the CTM-MTB assay.

Altogether, Xpert MTB/RIF tested 50 samples MTBC positive. Of these, it reported two (4%) specimens containing RMP-resistant *M*. *tuberculosis* and 48 (96%) susceptible ones. Phenotypic DST confirmed all susceptibilities and resistances reported by Xpert MTB/RIF.

CTM-MTB yielded the highest rate of invalid results (5/121; 4.1%); i.e. three out of the sub-group of TB-samples and two non-TB samples (Table 1). All samples appeared to be invalid due to presence of inhibitors since valid results were obtained after dilution (1:10) of samples (data not included). Only one (0.8%) DTB test showed inhibition; Xpert MTB/RIF produced one (0.8%) sample classified “no result” (aborted test).

Hands-on time to process specimens and perform fully automated real-time PCR with Xpert MTB/RIF was less than 3 min per specimen with a turn-around time (TAT) of less than 2 hours. Xpert MTB/RIF uses individual cartridges allowing on-demand diagnostics which is very helpful especially for small numbers of tests. However, dealing with larger series, hands-on time per test cannot be significantly reduced. In contrast, CTM-MTB and DTB are run in batch mode processing up to 48 and 96 samples, respectively. CTM-MTB is performed in three steps comprising specimen processing, preparation of PCR mixes and real-time PCR adding up to a hands-on time of approximately 40 min for 30 specimens in our laboratory. The DTB assay requires multiple work-stations for specimen processing, priming, pre-amplification prior to automated SDA in the ProbeTec ET instrument adding up to a hands-on time of 50 min for 30 specimens in our lab. Thus, hands-on times per test were in a similar range while total TATs were substantially longer compared to Xpert (DTB: 3 h 40 min; CTM-MTB: 4 h). Nevertheless, both assays, DTB and CTM-MTB can easily produce results within one working day.

## Discussion

Our study is the first one presenting a head-to-head comparison of Xpert MTB/RIF with two other widely used, commercial assays for detection of MTBC in clinical specimens. The study was designed for this comparison particularly addressing sensitivity in paucibacillary specimens such as culture-positive/smear-negative specimens and specificity in smear positive specimens with NTM. Therefore, these two groups were purposely over-represented in the study set (42.1% and 19.8%, respectively) compared to routine diagnostic setting where we found for example only 378 (1.8%) smear-negative/culture-positive for MTBC, and 86 (0.4%) smear positive specimens with NTM in a total number of 20,887 diagnostic specimens in our laboratory in 2011. This means that overall sensitivity and specificity values as well as positive and negative predictive values need to be re-calculated for the situation of routine diagnostics.

Besides detection of MTBC, the accurate discrimination of MTBC from NTM is becoming more important, not only in industrialized countries [17,18] but also in regions of Africa where NTM are often isolated from sputum cultures [9,19]. We have, therefore, included 24 (26% of culture positives) samples from which grew NTM in order to have a representative number of such samples for the comparison of specificities.

The sensitivity of 68% found for Xpert MTB/RIF with culture positive/smear-negative TB samples was in good concordance with data reported by other studies (range: 57.1% - 76.9%, mean: 68.0%) [1,3,9,10,12,14]. However, sensitivity for smear-positive TB samples was only 94.1% compared to a mean value of 98.8% (range: 97.7% - 100%) reported by other authors [1,3,9,10,12,14]. This could be partly explained by potential limitations of the specimens used for our investigation. The material had been frozen at −80°C for up to four years. After thawing it was diluted approximately 1.5 fold and aliquotted. Both, freezing and initial dilution might have affected sensitivity in a negative manner compared to fresh, undiluted material. On the other hand, similar sensitivities for fresh and frozen material, respectively, have been shown in analytical studies on Xpert MTB/RIF [2]. Several other studies that have used frozen specimens found a high level of performance for Xpert MTB/RIF [9,11,14] suggesting a limited impact of freezing on test sensitivity.

Overall sensitivity of 74.6% for Xpert MTB/RIF was in the lower range of values reported in the literature for respiratory specimens [1,3,9,10,12,14] (78.% – 92.2%; mean: 88%). The likely reason for this discrepancy is the low proportion of smear-positive *vs*. smear-negative TB samples (ratio 0.33) biasing overall sensitivity in a negative manner. For Xpert MTB/RIF, most of other evaluation studies used sample sets with ratios of smear-positive to smear-negative specimens of ≥ 2 [1,3,9,12].

Whereas we recorded excellent specificity (100%) of Xpert MTB/RIF with culture-negative specimens, we found poor specificity (91.6%) within the sub-set of NTM samples. Substantial numbers of samples with NTM have been included only in few other evaluation studies; the studies by Moure et al. [11] and Marlowe et al. [10] reported 100% specificity with 20 and 41 NTM samples, respectively, whereas Rachow et al. [9] found one of 45 patients with NTM tested positive by Xpert MTB/RIF resulting in a specificity of 97.8%. A recent analytical study detected no cross-reactivities with NTM species except some weak cross-hybridization of two *rpoB* specific molecular beacons with *M*. *malmoense*[2]. The reason for two false-positive MTBC signals in our study, albeit classified as “low”, remains unclear. One specimen was recovered from a patient with repeated isolation of *M*. *kansasii* and known NTM infection; the second one from a patient with *M*. *avium* infection. In neither case, MTBC has ever been isolated from clinical specimens. Furthermore, both other tests yielded negative results thereby making the presence of DNA from MTBC unlikely.

Compared to Xpert MTB/RIF, DTB showed slightly higher sensitivity rates for smear-positive (100% *vs*. 94.1%; *P* = 0.5) as well as for smear-negative TB samples (72% *vs*. 68%; *P* = 0.66), although these differences were not significant. These are the very first head to head comparison data of Xpert MTB/RIF and DTB. So far, several evaluation studies [1,3] have attested Xpert MTB/RIF a remarkably high sensitivity due to the heminested real-time PCR technology, detecting down to ~ 130 CFU per ml specimens [2]. Our findings might be explained by a certain benefit from the multi-copy target IS*6110* used by DTB [14]. Lowest sensitivity values were obtained with the CTM-MTB assays. Relatively low sensitivity of the CTM-MTB for smear-negative samples is a known shortcoming and has been recently confirmed by direct comparison to Xpert MTB/RIF using non-respiratory specimens (78% *vs*. 98%) [13]. On the other hand, CTM-MTB performed best in terms of specificity, and was the only assay yielding 100% specificity in our investigation. Compared to CTM-MTB, the specificity values of DTB (98.1%) and Xpert MTB/RIF (96.2%) were lower due to false positive results with NTM samples, although differences were not significant (*P* = 0.5 and *P* = 0.25, respectively). For DTB, some cross-reactivity between the target (IS*6110*) and NTM has been reported earlier [20], and was mostly associated with low MOTA values. This applied also to the false-positive case in our study. DTB might be generally more prone to unspecific reactions in the “low-positive zone” than e.g. CTM-MTB [20,21] which can be mostly overcome by re-testing the sample.

Demands on NAATs are different in high and low prevalence settings and can be best reflected by the predictive values of the tests. Both, positive and negative predictive values are directly depending on the prevalence of TB positive samples in the diagnostic collective of the laboratory. In a high prevalence setting, where e.g. up to 25% of specimens are expected being TB positive, a minor increase of sensitivity of few percent points can significantly increase the negative predictive value while the influence of the specificity on the positive predictive value is comparably low. In these circumstances, a specificity of over 99% is desirable, but not essential: a decrease from 99% to 97% would only decrease the positive predictive values from 97% to 91.7%, when e.g. calculated for a prevalence of 25% TB positive samples. The performance characteristics of Xpert MTB/RIF would perfectly match these requirements. In contrast, in a low prevalence setting (5% TB positive samples) a low specificity of 97% would have a dramatic impact on PPV lowering it to 63.7%. Furthermore, in countries with a high percentage of NTM isolates a high specificity value is indispensible in order to discriminate tuberculous from non-tuberculous bacteria in smear-positive samples. In this respect, the CTM-MTB seems being slightly superior to the other assays. However, its relative complexity, the requirement of pre-amplification areas and the need of staff experienced with molecular biology methods makes it suitable mainly for modern molecular microbiology laboratories with high capacity diagnostics. In contrast, the Xpert MTB/RIF is a self-contained, integrated test which requires hardly any training and can be also used in resource-constrained, low technology settings.

## Conclusions

In summary, the three assays yielded good and comparable performance characteristic in terms of sensitivity and specificity. Xpert MTB/RIF had a performance similar to that of DTB with some weakness regarding specificity particularly within the disproportionately large group of NTM samples. CTM-MTB showed the highest specificity of all potentially yielding the best positive predictive value for daily routine TB diagnostics in low prevalence settings.

## Competing interests

The authors declare that they have no competing interests.

## Authors’ contributions

UA participated in the study design, organized and supervised the laboratory work and analysed the data. SH-T participated in data analysis and drafted the manuscript. LT, AE, MA, ES and TA did most of the experimental work. HH supervised the study and participated in preparation of the manuscript. All authors read and approved the final manuscript.

## Pre-publication history

The pre-publication history for this paper can be accessed here:

http://www.biomedcentral.com/1471-2334/13/280/prepub
